# The Altheat Stove

**Published:** 1914-02-14

**Authors:** Ethel Lewis

**Affiliations:** 7 Auckland Road, Upper Norwood, S.E.


### The Altheat Stove.

*To the Editor of* The Hospital.

Sir,—I am shortly starting a nursing home in Kensington, and am desirous of finding the best gas-stove for heating purposes. Some months ago I saw a notice in The Hospital of the Altheat stove.

Knowing the great interest you take in nursing matters, I thought perhaps you could inform me whether the Altheat stove has been proved a satisfactory one for use in a sick-room.

I should esteem it a great favour if you will give me information concerning it.—I am, yours faithfully,

(Miss) Ethel Lewis.

7 Auckland Road, Upper Norwood, S.E.,

February 5, 1914.

[The Altheat stove is the invention, we learn, of a member of the profession. We have given it a prolonged trial in the sick-room, and also in bed and sitting rooms, and the reports have been excellent. There has been all through : No smell, no unpleasantness, nor any irritating dryness in the air. The heat the stove gives is plentiful, and its volume can be easily regulated to meet all circumstances. It is the first gas-stove we have found that makes heating by gas in bedrooms entirely satisfactory.—Ed. The Hospital.]

